# Reducing the number of CTs performed to monitor personalized dosimetry during peptide receptor radionuclide therapy (PRRT)

**DOI:** 10.1186/s40658-018-0211-1

**Published:** 2018-06-19

**Authors:** Alexandre Chicheportiche, Faozi Artoul, Arnon Schwartz, Simona Grozinsky-Glasberg, Amichay Meirovitz, David J. Gross, Jeremy Godefroy

**Affiliations:** 1 Department of Nuclear Medicine and Biophysics, Hadassah-Hebrew University Medical Center, 91120 Jerusalem, Israel; 2 Neuroendocrine Tumor Unit, Endocrinology and Metabolism Department, Hadassah-Hebrew University Medical Center, 91120 Jerusalem, Israel; 3 Oncology Department and Radiation Therapy Unit, Hadassah-Hebrew University Medical Center, 91120 Jerusalem, Israel

**Keywords:** Peptide receptor radionuclide therapy (PRRT), [^177^Lu]-DOTA-TATE, Dosimetry, Kidneys, Bone marrow, Single photon emission computed tomography (SPECT), Computed tomography (CT)

## Abstract

**Background:**

Peptide receptor radionuclide therapy (PRRT) with [^177^Lu]-DOTA-TATE is an effective treatment of neuroendocrine tumors (NETs). After each cycle of treatment, patient dosimetry evaluates the radiation dose to the risk organs, kidneys, and bone marrow, the most radiosensitive tissues. Absorbed doses are calculated from the radioactivity in the blood and from single photon emission computed tomography (SPECT) images corrected by computed tomography (CT) acquired after each course of treatment. The aim of this work is to assess whether the dosimetry along all treatment cycles can be calculated using a single CT. We hypothesize that the absorbed doses to the risk organs calculated with a single CT will be accurate enough to correctly manage the patients, i.e., whether or not to continue PRRT.

Twenty-four patients diagnosed with metastatic NETs undergoing PRRT with [^177^Lu]-DOTA-TATE were retrospectively included in this study. We compared radiation doses to the kidneys and bone marrow using two protocols. In the “classical” one, dosimetry is calculated based on a SPECT and a CT after each treatment cycle. In the new protocol, dosimetry is calculated based on a SPECT study after each cycle but with the first acquired CT for all cycles.

**Results:**

The decision whether or not to stop PRRT because of unsafe absorbed dose to the risk organs would have been the same had the classical or the new protocol been used. The agreement between the cumulative doses to the kidneys and bone marrow obtained from the two protocols was excellent with Pearson’s correlation coefficients *r* = 0.95 and *r* = 0.99 (*P* < 0.0001) and mean relative differences of 5.30 ± 6.20% and 0.48 ± 4.88%, respectively.

**Conclusions:**

Dosimetry calculations for a given patient can be done using a single CT registered to serial SPECTs. This new protocol reduces the need for a hybrid camera in the follow-up of patients receiving [^177^Lu]-DOTA-TATE.

## Background

^177^Lutetium (^177^Lu) is a useful radionuclide in several targeted peptide receptor radionuclide therapies (PRRTs) due to its favorable decay characteristics and the possibility of reliable labeling of biomolecules used for tumor targeting. Being both a beta and gamma emitter, the therapeutic effect achieved thanks to beta emission can be monitored by a gamma camera with planar or SPECT imaging. In order to maximize the treatment efficiency of [^177^Lu]-DOTA-TATE [1–3], shown to be an effective therapy of neuroendocrine tumors (NETs), the amount of ^177^Lu radioactivity to be administered has to achieve an optimal therapeutic effect of radioligand therapy, i.e., to lead to a maximal absorbed dose in the tumors with limited side effects to the most radiosensitive non-pathological tissues, namely the kidneys and bone marrow [4–7]. For this purpose, individual dosimetry after each cycle of treatment is necessary to evaluate the cumulative absorbed dose to the organs at risk and to decide whether the patient can continue to receive further treatments. Recommended schedule of treatment with [^177^Lu]-DOTA-TATE [8] consists of a fractionated therapy of four 7.4 GBq (200 mCi) injections spaced by 6–12 weeks, provided that the cumulative dose will not exceed the safety limits of 23 Gy [9–11] for the kidneys and 2 Gy [9, 12] for the bone marrow. It has previously been shown that in most cases, the kidneys are the dose-limiting organs in PRRT treatments with [^177^Lu]-DOTA-TATE [9]. However, a recent study by Bergsma et al. [13] showed low nephrotoxicity after PRRT with a cumulative activity of 29.6 GBq (800 mCi) of [^177^Lu]-DOTA-TATE and a mean calculated radiation dose to the kidneys of 20.1 ± 4.9 Gy. Three of their 323 patients developed renal toxicity grade 2, and no grade 3 or 4 renal toxicity occurred. Other authors [7, 14] have argued that higher safe absorbed doses to the kidneys (28–29 Gy) could probably be adopted due to the inhomogeneous distribution at a microscopic level of PRRT with [^177^Lu]-DOTA-TATE and to the short range of its β-particles in the tissue.

In order to estimate the pharmacokinetics of [^177^Lu]-DOTA-TATE and to calculate the radiation doses absorbed by the patient’s organs, quantitative single photon emission computed tomography (SPECT) images corrected for photon attenuation (from computed tomography (CT) attenuation maps) and blurring (resolution recovery) are acquired after each cycle of treatment [15, 16]. Following the EANM/MIRD guidelines [17], full dosimetry is performed after the first treatment with three SPECT/CT acquisitions at 24, 96, and 168 h after the administration of [^177^Lu]-DOTA-TATE. For the subsequent treatment cycles, only a single SPECT/CT study approximately 24 h after the treatment is performed assuming an unchanged effective half-life of [^177^Lu]-DOTA-TATE between treatments as proved and proposed by Garske et al. [12]. In our study, the radioactivity distribution and pharmacokinetics of [^177^Lu]-DOTA-TATE in the kidneys, liver, spleen, remainder of the body, and tumors have been estimated by drawing volumes of interests (VOIs) either in the anatomical (CT) or functional (SPECT) images. The kinetics of radioligand in the bone marrow has been estimated from the radioactivity concentration measured in blood samples taken after the treatment. Indeed, Forrer et al. [18] showed that the activity concentration in the bone marrow after the treatment is equal to that in the blood.

A typical patient, completing 4 cycles of treatment, would have done six SPECT/CTs across his whole PRRT treatment. Across the different cycles of treatment, the acquired CT may show changes that can be caused by the changes in the patient position under the gamma camera, response to treatment or progressive disease, change in patient weight, or gastric emptiness or fullness. However, due to the short time interval between courses of treatment and the usually slow response to treatment (or alternatively disease progression) in a population of patients receiving PRRT, our impression was that in most cases, no visually blatant changes were observed on the CTs between treatments. Therefore, for a given patient, attenuation maps based on subsequent CTs across the cycles of treatment should be very similar. Consequently, we hypothesize that the use of the attenuation map generated from the CT of the first SPECT/CT acquisition provides a sufficiently good match to be used for subsequent SPECTs.

The main aim of this work was to compare, over the whole series of treatment, the dosimetry results for the kidneys and bone marrow based on the “classical” protocol to those based on a new protocol. In the “classical” one, dosimetry is calculated based on a SPECT and CT after each cycle. In the new one, the dosimetry is calculated based on a SPECT after each cycle of treatment but on one single CT for all the cycles. Individual patient dosimetry along the treatment is used to design a patient-tailored treatment schedule. At our institution, before giving a subsequent PRRT treatment, the absorbed doses to the kidneys and bone marrow during the previous *p* treatments are examined and an expected cumulative dose after the following (*p* + 1) cycle is determined. The latter is calculated as the mean dose over the previous *p* treatments to which is added the cumulative dose absorbed over these *p* treatments. In our institution, we withheld PRRT if the expected cumulated dose after the next treatment will exceed by 10% the safe dose limits, i.e., 25.3 Gy for the kidneys and 2.2 Gy for the bone marrow (except for very specific clinical setups where cost/benefit ratio led us to continue treatment beyond these limits). In this work, we compared the actual patient management (whether or not treatment was stopped due to dosimetry) to the hypothetical management of the patient, had we used the new “single CT” protocol.

Percentage differences in the absorbed doses calculated using the two protocols will be shown on Bland and Altman [19] plots for the kidneys and bone marrow. Also, Pearson’s correlation coefficient between data obtained from one method versus another has been computed and will be presented for the two risk organs. Finally, the intra- and inter-observer reproducibility will be also calculated.

## Methods

### Patients

Between March 1, 2015, and July 1, 2016, 160 PRRT treatment cycles with [^177^Lu]-DOTA-TATE were administered to 64 patients at our institution. Inclusion criteria for this study were (a) age ≥ 20 years, (b) patients who started and completed their series of treatments in this period, and (c) patients for whom the sole reason for treatment discontinuation was treatment toxicity (hematotoxicity, although not reflected by the bone marrow dosimetry or general deterioration) or an expected absorbed dose > 25.3 Gy to the kidneys and > 2.2 Gy to the bone marrow.

**Fig. 1:**
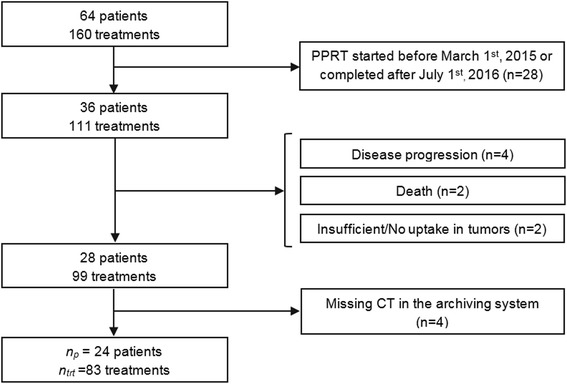
Chart of patient inclusion. *n*_*p*_ represents the number of patients included in the study, and *n*_*trt*_ is the corresponding total number of therapy cycles

Among these 64 patients, 36 of them started and completed their therapy between March 2015 and July 2016. In total, four patients were excluded because of disease progression and two were deceased before completing the series of treatment. Two patients who received a single treatment because of an insufficient or inexistent uptake in tumors were also excluded from the study. Finally, four more patients were excluded due to missing data on hospital archiving system. After all, 24 patients (12 men, 12 women; average age 61 years, range 37–85 years) were included in this single-center retrospective study (Fig. 1). The total number of therapy cycles *n*_*trt*_ included in the study is 83 with a median equal to four (1 to 4 cycles).

Patient demographics are shown in Table 1.

**Table 1:** Demographic data

Characteristic	Value
Total number of patients	24
Age (years)	
Mean ± standard deviation	61 ± 13
Range	37–85
Gender	
Male	12
Female	12
Primary tumor site	
Pancreas	12
Stomach	1
Skin	1
Intestine	1
Colon	3
Rectum	1
Lung	3
Unknown	2
Sites of metastases	
Liver	22
Lymph nodes	8
Bone	5
Lung	2
Peritoneum	3
Pancreatic bed	1

### PRRT therapy

[DOTA^0^,Tyr^3^] Octreotate was obtained either from ABX (Radeberg, Germany) or CS Bio Co. (Menlo Park, CA, USA). ^177^LuCl_3_ was supplied by PerkinElmer, Inc. (Waltham, MA, USA) and [^177^Lu]-DOTA-Octreotate was locally prepared by Isorad Ltd. (Soreq NRC, Yavne, Israel). Quality control for radiochemical purity was performed on each lot using high-performance liquid chromatography (HPLC) and instant thin-layer chromatography (ITLC) scanner and only labeling yields over 99% were accepted for treatment.

Infusion of amino acids (Vamin 18 g N/L electrolyte-free, Fresenius Kabi) started about 1.5 h before the administration of the radiopharmaceutical and lasted for 5 h. The radioactive ligand, diluted in 500 ml of saline, was co-administered intravenously over a period of 30 min. The mean activity per cycle of treatment was 7.4 ± 0.25 GBq (200.4 ± 6.7 mCi) with a median cumulative activity of 29.2 GBq (7.5–30.4 GBq). The interval between treatment cycles was 6–12 weeks.

### Image acquisition

All 24 patients included in the study underwent a planar whole-body examination under a gamma camera after each cycle of treatment. Additionally, SPECT/CTs of the abdomen including the kidneys, liver, and spleen were acquired 18 h, 25 h, and 7 days after the injection of the first therapeutic dose in order to estimate the pharmacokinetics of [^177^Lu]-DOTA-TATE in these organs. For the following treatments, patients underwent a single SPECT/CT about 20 h after the administration of the radiopharmaceutical, assuming minor changes in the effective half-life for organs of interest [12]. All images were acquired on a Discovery NM/CT 670 camera with anatomical image capability (International General Electric, General Electric Medical Systems, Haifa, Israel). This system combines a dual-head coincidence SPECT camera with an axial field of view (FOV) of 40 × 54 cm, a NaI(Tl) crystal thickness of 9.5 mm, and 59 photomultiplier tubes (PMT). All functional images were acquired with a 20% energy window around the main photopeak of ^177^Lu (208 keV; 11% probability) [20] with medium-energy general purpose (MEGP) collimators. Whole body images were acquired with step-and-shoot mode (180 s per view; about 20 min acquisition) in a 256 × 1024 matrix, zoom 1.0, and body contour. SPECT imaging was performed applying 60 views over 360° (30 angular steps per head, 6° angle step) with a 30 s exposure per frame (15 min acquisition) in a 128 × 128 matrix size (4.4 mm pixels), zoom 1.0, and body contour. Anatomical CT images were acquired before each SPECT acquisition with the integrated BrightSpeed multidetector CT (24 rows – maximum 16 slices/rotation) using a tube voltage of 120 kV and the smart current option (80–220 mA). Calibration of SPECT images was based on a series of 29 SPECT acquisitions of a 20-mL vial placed in the center of the gamma camera FOV with a known activity of ^177^Lu ranging from 114.7 MBq (3.1 mCi) to 7215 MBq (195 mCi). The ^177^Lu calibration source was placed in the center of eight 1-L saline bags with two additional distant 20-mL ^177^Lu sources in order to simulate an amount of scatter similar to a clinical scan. However, no scatter correction was applied, and thus, contributions from scattered photons were ignored. No dead time was observed during calibration. This study was performed entirely independently of the camera vendor.

### Blood activity concentration measurements

In order to quantify the self-dose to the bone marrow, blood samples were drawn at 18 and 25 h after the first injection of the radiopharmaceutical and after about 20 h for the following treatments. No other samples have been drawn subsequently for all the 24 patients included in this work due to organizational constraints in our department.

The samples were accurately weighed (Sartorius BL310 balance, 0.01 g precision), and the radionuclide activity concentration in the blood was measured using a NaI(Tl) well gamma-counter (Wizard 1480 3″, Perkin Elmer). The measurements were repeated three times for each blood sample. Normalization of the counter was made every 6 months, unless the instrument sends a warning for performing it. The blood activity concentration has been fitted by a mono-exponential curve and integrated to infinity in order to estimate the residence time and then the self-absorbed dose to the bone marrow, assuming that the activity concentration in the latter is the same as in the blood [18].

### Image analysis and dosimetry calculation

**Fig. 2:**
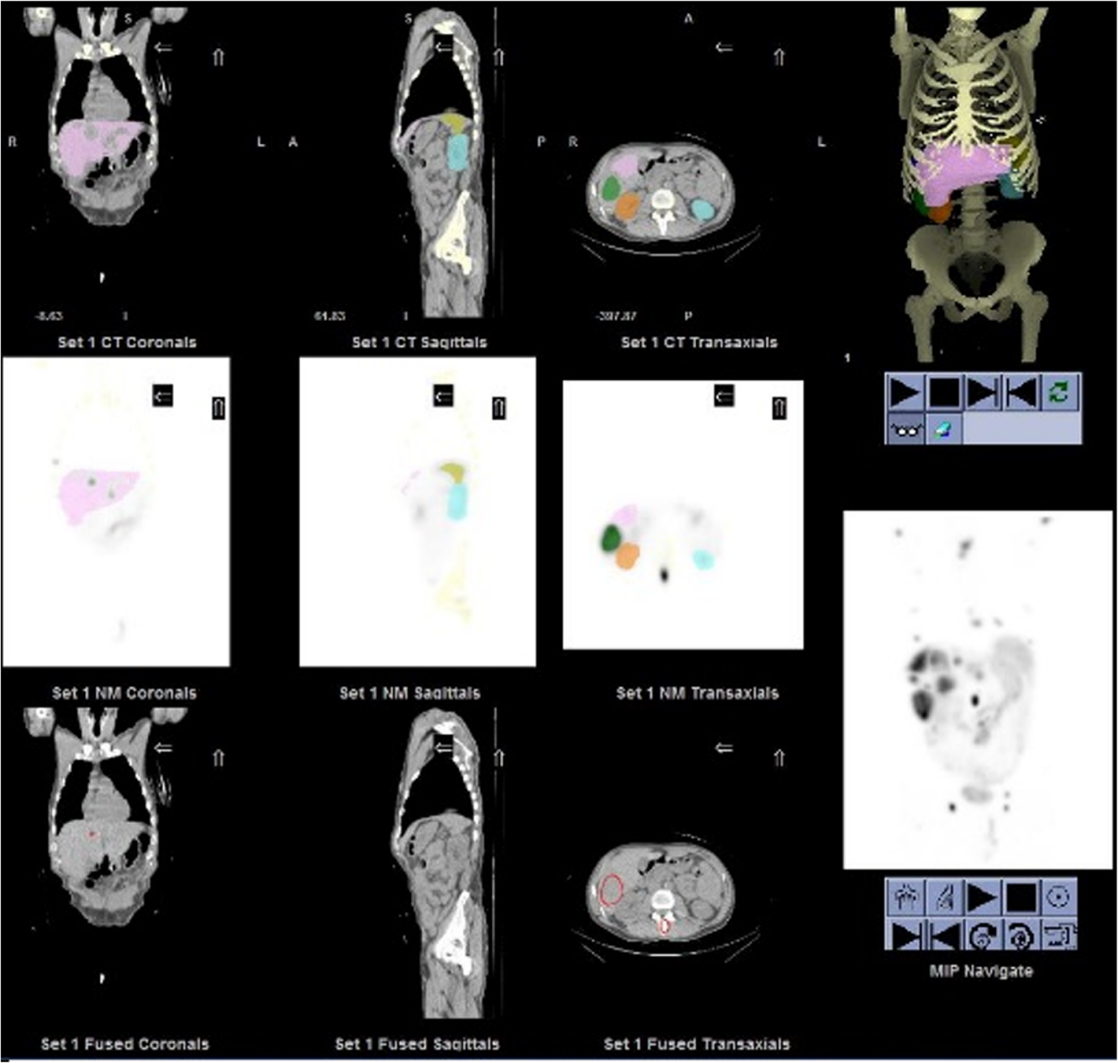
Volume of interest (VOIs) of the kidneys, liver, spleen, and tumors drawn using the General Electric Dosimetry toolkit software

Image analysis for dosimetry was performed using the General Electric (GE) Dosimetry toolkit (DTK) software [21] available for the Xeleris 3.0 Workstation (International General Electric, General Electric Medical Systems, Haifa, Israel). The ordered subsets expectation maximization (OSEM) algorithm with attenuation correction (from CT attenuation maps) and resolution recovery (for blurring) included in the Xeleris 3.0 workstation were used. No scatter correction was applied. In the current processing, CT is used for attenuation correction and for two types of registration: (i) registration of SPECT with CT and (ii) registration of the three SPECT/CTs (of the first cycle of treatment) one with the other. This allows the transfer of the VOIs drawn on the first SPECT/CT to the two other. DTK proposes different types of automatic or semi-automatic registrations: SPECT/CT “inherently aligned,” alignment “by center of volumes” (force the center of the SPECT raster to match the center of the CT raster), alignment “by table position” (adjust automatically according to table position during the SPECT and the CT acquisition), or alignment “by single landmark” (the user mark one spot in each modality and the rest of the volumes are registered accordingly). An additional option allows also manual adjustments of the alignment in the three dimensions (roll, pan, azimuth, and elevation) by the user. However, none of the automatic or semi-automatic registrations led to a correct registration between the first acquired CT and a SPECT acquired at a different date. Therefore, all the registrations between the first CT and the subsequent SPECTs (the first one excluded) were done manually. Using the “classical” protocol, SPECT and CT acquisitions were inherently aligned and did not need any additional adjustment. Processing includes either semi-automatic (threshold approach) or manual three-dimensional delineation of the organs on functional (SPECT) or anatomical (CT) images. VOIs were placed over the whole healthy organs of interest (kidneys, liver, spleen, and remainder of the body) and over tumors. For the kidneys and spleen, VOIs have been drawn using either the semi-automatic delineation tool on SPECT images or manual delineation on CT. The delineation method used was user- and case-dependent; in cases where the kidneys/spleen was well defined in SPECT images without surrounding tumors, the semi-automatic delineation tool has been used while in other cases, a manual delineation has been performed. It should be noted though that even when a semi-automatic method was used, the threshold of uptake for delineation was defined anew for each patient, thus increasing its user dependency. For the healthy liver and remainder of the body, VOIs have been drawn using manual delineation for all the 83 treatment cycles included in this study. For tumors, SPECT-based semi-automated segmentation has been used. Figure 2 shows an example of the drawn VOIs using the GE DTK software. It is noteworthy that the GE DTK software does not allow copying the VOIs delineated on a SPECT/CT from 1 cycle of treatment to the SPECT/CT done after another cycle of treatment. Thus, VOIs were drawn again on attenuation corrected SPECT images (using either the “classical” or the “single CT” protocol) for each cycle of treatment. Finally, the GE DTK software gives the volume of the VOIs and the activity concentrations in the organs of interest and tumors for each time point (18 h, 25 h, and 7 days post-injection for the first cycle and 20 h post-injection for the following ones). An in-house Interactive Data Language (IDL) code developed in our department is then used to obtain the absorbed radiation doses.

After the first treatment, for the kidneys, liver, spleen, remainder of the body, and tumors, residence times have been calculated as the area under the curve of a single exponential fit of the activity concentrations values given by GE DTK software at 18 h, 25 h, and 7 days post-injection. For the blood, the mono-exponential fit is based only on values at 18 and 25 h post-injection. For the following treatments, assuming an unchanged effective half-life of [^177^Lu]-DOTA-TATE for these organs, the activity concentrations at different times are deduced [12]. Radiation absorbed doses to the tumors were computed by using the method proposed by Sandström et al. [22] where self-doses only are taken into account. The absorbed doses (mGy) were obtained by the multiplication of the residence time of the radioactivity concentration in the tumor ([MBq ⋅ s]/[MBq ⋅ kg]) by an appropriate dose concentration factor (DCF_tumor_ = 0.0236 [mGy ⋅ g]/[MBq ⋅ s]) and by the administered activity *A*_adm_ (MBq). Radiation absorbed doses to the kidneys, liver, spleen, bone marrow, and remainder of the body were calculated using the Medical Internal Radiation Dose (MIRD) formalism [16] where self-doses and cross-doses are considered as follows:

1 $$ D\left({r}_{\mathrm{t}}\right)={A}_{\mathrm{adm}}\bullet \left[{t}_{{\mathrm{r}}_{\mathrm{t}}}\bullet \mathrm{DF}\left({r}_{\mathrm{t}}\leftarrow {r}_{\mathrm{t}}\right)+\sum \limits_{\mathrm{s}\ne \mathrm{t}}{t}_{{\mathrm{r}}_{\mathrm{s}}}\bullet \mathrm{DF}\left({r}_{\mathrm{t}}\leftarrow {r}_{\mathrm{s}}\right)\right] $$

with *D*(*r*_t_) the dose absorbed in the target organ *r*_t_ in [mGy]; ${t}_{{\mathrm{r}}_{\mathrm{t}}}$ and ${t}_{{\mathrm{r}}_{\mathrm{s}}}$ respectively the residence times in the target *r*_t_ and source organ *r*_s_ in [MBq∙s]/[MBq∙kg], and DF(*b*←*a*) the dose factor for a couple source *a*-target *b* in [mGy]/[MBq∙s]. The DFs were taken from OLINDA/EXM 1.0 [23] for the adult male and adult female phantoms. The residence times for a specific organ have been scaled for differences in mass between the drawn VOIs (considering a tissue density of 1.0 g/cm^3^) and the mass organ considered in OLINDA/EXM 1.0 [24]. Because most patients had a significant uptake in tumors, the latter have been taken into account for the cross-dose calculation to other organs. The DFs used for tumors are those of the liver since tumors are generally localized there. For the remainder of the body, the cross-dose contribution to the bone marrow is overestimated in OLINDA/EXM 1.0 when there is no uptake of the radionuclide in the bone, as shown previously by Stabin et al. [25]. Thus, the cross-dose DF from the remainder of the body to the bone marrow has been modified and replaced by a DF equal to 30.3 nGy∙kg/MBq∙s for the male adult phantom and 35.8 nGy∙kg/MBq∙s for the female adult phantom as given in Ref. [26].

In this work, only the absorbed doses to the kidneys and bone marrow obtained with the “classical” and “single CT” protocols are presented and compared since the dose to other organs does not influence the patient management.

### Statistical methods

Absorbed dose results to the kidneys and bone marrow were analyzed in two ways. First, the Bland and Altman plots showing differences between the results obtained from the “classical” and the “single CT” protocols were built. These plots show the absolute value of the difference between results obtained with the two protocols divided by the mean of both measurements versus the mean of the measurements. Secondly, the Pearson’s correlation coefficient between data obtained from one method versus another has been computed.

Intra-observer reproducibility was determined for all the 24 patients for the kidneys and bone marrow using Bland and Altman plots and linear regression analysis to assess the intrinsic consistency of each measurement. Inter-observer reproducibility was based on the analysis of two independent series of measurements made by two examiners. Intra- and inter-observer reproducibility were estimated from measurements using the “classical” protocol in order to exclude the influence of suboptimal co-registration of SPECTs and CTs. Indeed, as said before, using the classical protocol SPECT and CT acquisitions are inherently aligned such that the examiners did not need to do any adjustment for all the 24 patients.

For all tests, *P* < 0.01 was considered significant.

## Results

**Fig. 3:**
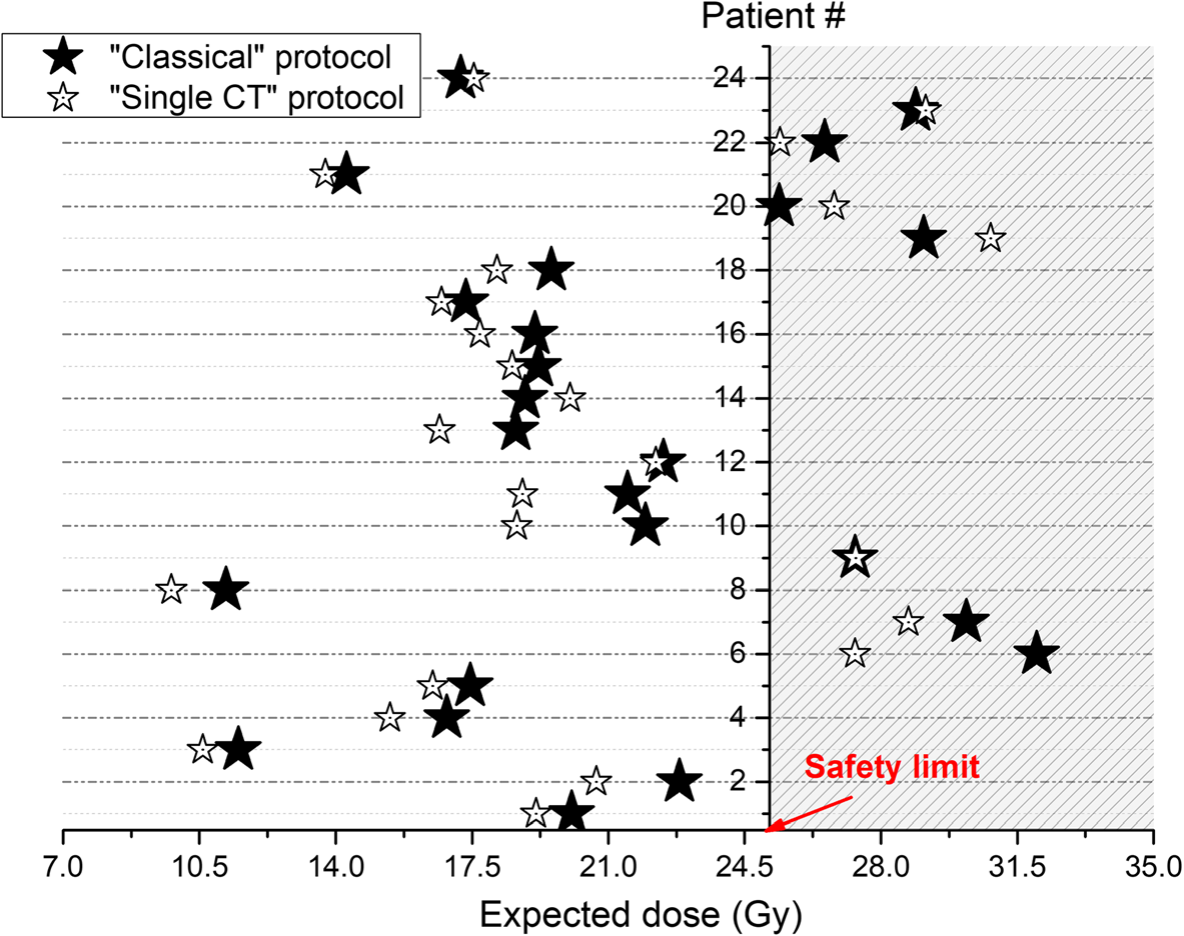
Expected dose after the last PRRT calculated from the previous ones (1 to 3) using the “classical” protocol (black stars) or the “single CT” protocol (empty stars). Each horizontal line represents a patient, and the dashed region represents the unsafe dose region and interruption of the treatment (patients # 6, 7, 9, 19, 20, 22, and 23)

Figure 3 shows the expected cumulative doses for kidneys calculated using either the “classical” or the “single CT” protocol. For all the 24 patients, the hypothetical management based on the new “single CT” protocol would have been similar to the “classical” management based on the whole series of SPECT/CTs as shown in this figure. Indeed, all the patients discontinued/not discontinued with the “classical” protocol would have been discontinued/not discontinued by using the new “single CT” protocol. Our null hypothesis was that there was no difference in the treatment management of the patients between the two protocols. A sample size of 24 patients has a power of 92% to detect a true difference of at least 10% at the 0.05 level of significance, using a one-sided binomial test. We used StatXact, Cytel Inc., Cambridge MA, version 10 20.

**Fig. 4:**
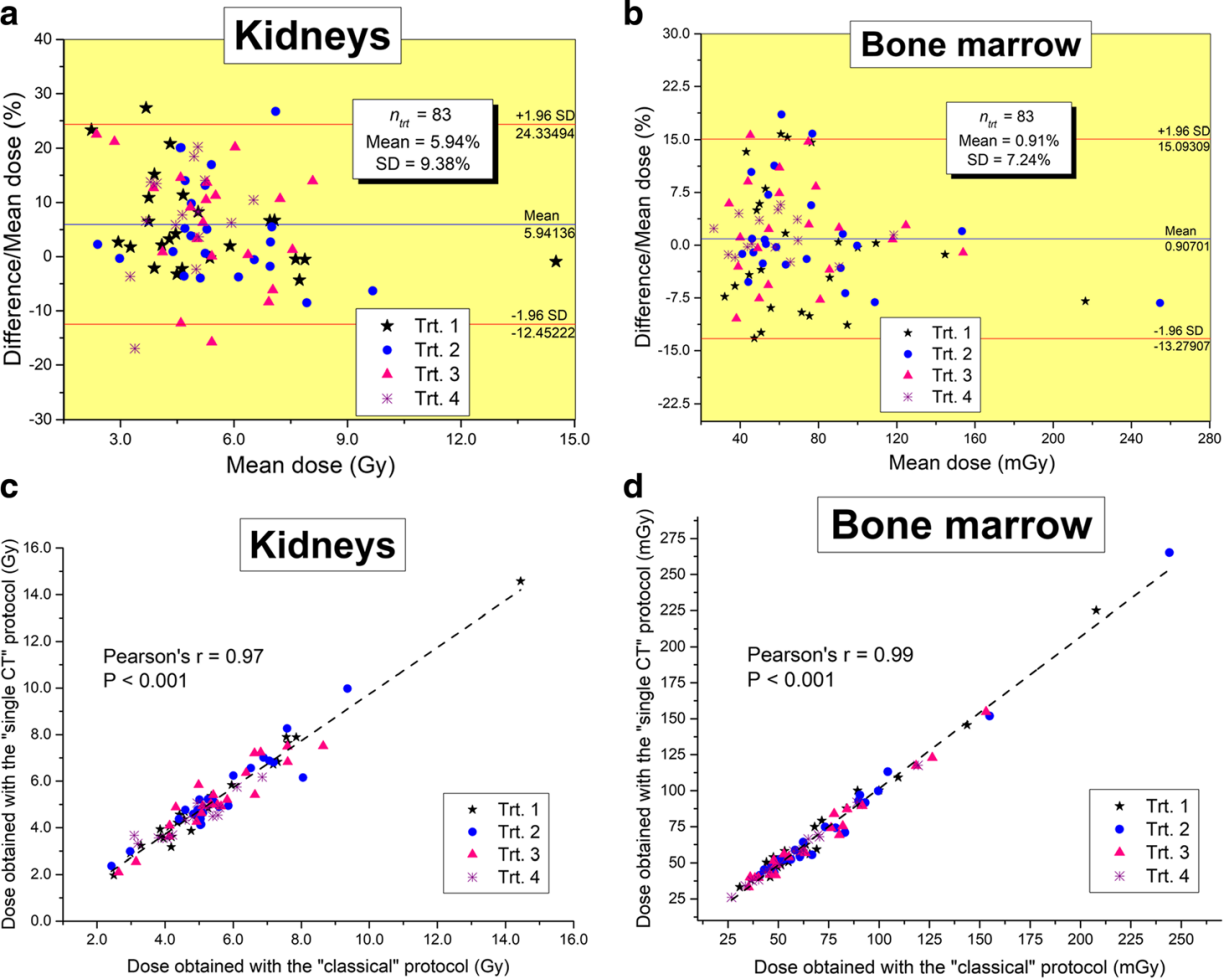
Bland and Altman plots showing differences in the absorbed doses to the kidneys (**a**) and bone marrow (**b**) after PRRT cycles 1 to 4, computed using the “classical” protocol and the “single CT” protocol, are presented with the 95% limits of agreement (mean ± 1.96 SD). Correlation plots for the doses obtained with the “single CT” protocol against the “classical” one are also shown for the kidneys in **c** and bone marrow **d**

**Fig. 5:**
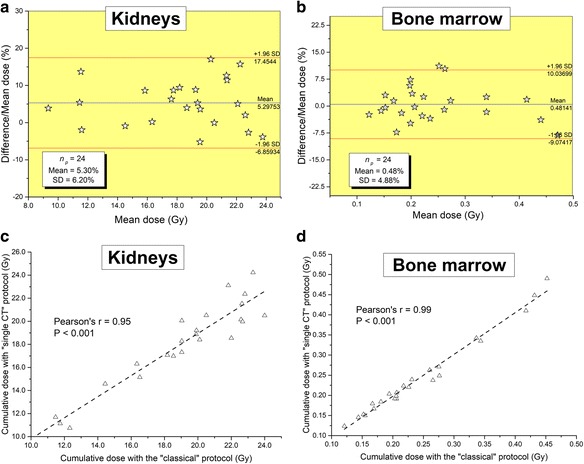
Bland and Altman plots showing differences in cumulative absorbed doses to the kidneys (**a**) and bone marrow (**b**) calculated using the “classical” protocol and the “single CT” protocol are shown with the 95% limits of agreement (mean ± 1.96 SD). Correlation plots for the cumulative doses obtained with the “single CT” protocol against the “classical” one are also shown for the kidneys in (**c**) and bone marrow in (**d**)

Figure 4 shows the differences in the absorbed doses to the kidneys and bone marrow calculated from the two different protocols for all the 83 treatments included in this study. The Bland and Altman plots, showing the relative difference between the two calculation methods, and the correlation curves, testifying to the degree of relationship between them, are plotted in this figure. Similarly, Fig. 5 presents the results for the cumulative dose absorbed to these organs at risk for the 24 patients included in this work. It is noteworthy that the agreement between the two methods was excellent over all series of therapy cycles (cf. Fig. 4). Indeed, for the kidneys, mean relative differences of 5.94 ± 9.38% over the 83 therapy cycles and 5.30 ± 6.20% over the 24 cumulative doses has been obtained with a Pearson’s correlation coefficient *r* = 0.97 and *r* = 0.95, respectively (all *P* < 0.0001). For the bone marrow, the absorbed doses have been obtained with mean relative differences of 0.91 ± 7.24% over all the therapy cycles and 0.48 ± 4.88% for the cumulative doses with *r* = 0.99 (all *P* < 0.0001). It is noteworthy from these results that for the bone marrow, the error due to the “single CT” protocol is, as expected, very low since the largest contribution is derived from the locally absorbed dose [9] that is calculated from the radioactivity measured in the blood samples [18]. However, the bone marrow is by far a lower risk organ than the kidneys. Indeed, in Reference [9], the bone marrow was the dose-limiting organ in only 1.5% of the patients.

**Fig. 6:**
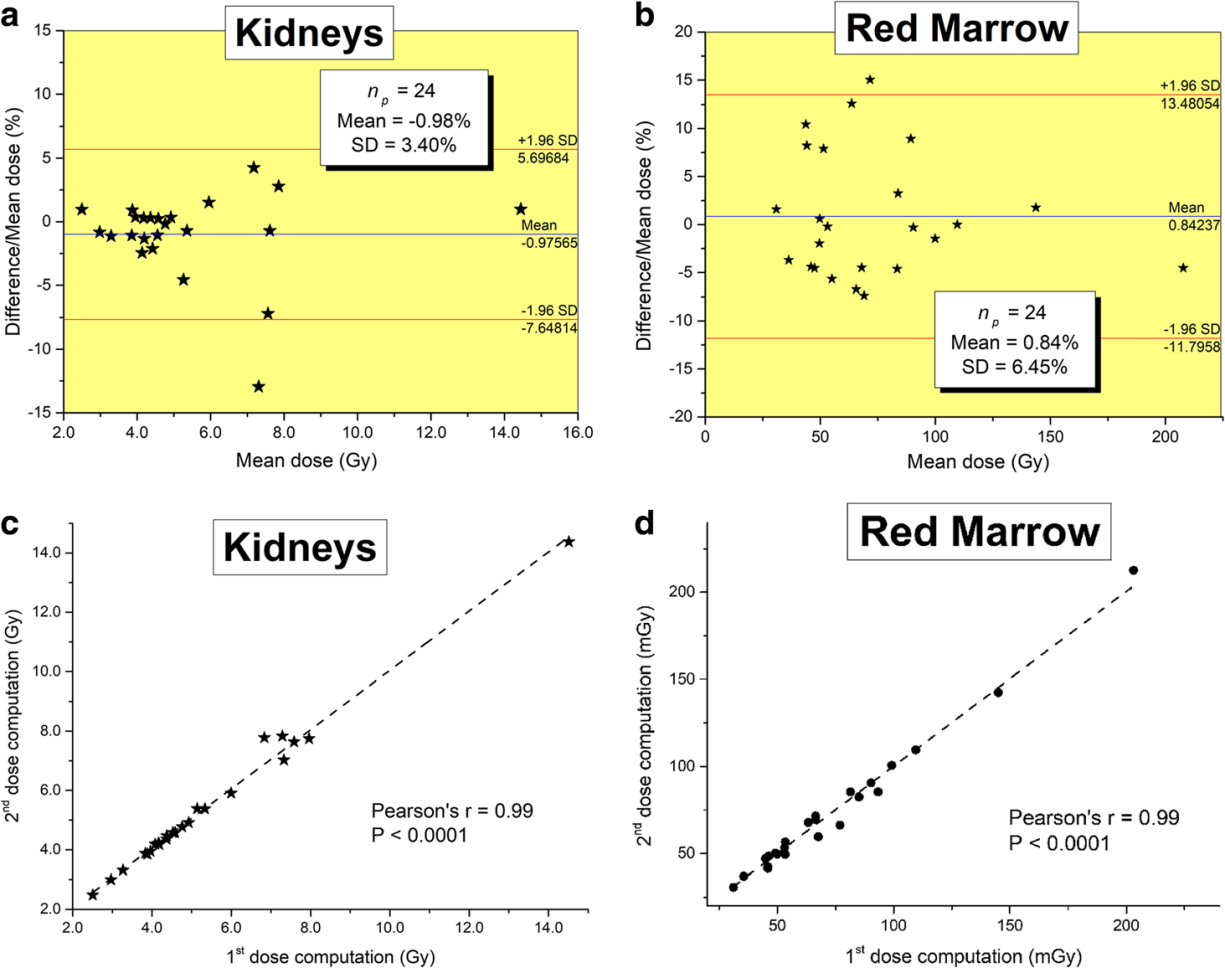
Bland and Altman plots showing differences in absorbed doses for a single therapy cycle to the kidneys (**a**) and bone marrow (**b**) calculated twice by the same user are presented with the 95% limits of agreement (mean ± 1.96 SD). Correlation plots obtained with the first measurement versus the second one are also shown for the kidneys in (**c**) and bone marrow in (**d**)

The intra-observer reproducibility for absorbed dose measurements was excellent for the kidneys and bone marrow (all *r* = 0.99 with *P* < 0.0001). The mean paired difference was − 0.98 ± 3.40% for the kidneys and 0.84 ± 6.45% for the bone marrow (cf. Fig. 6). Similarly, a good inter-observer agreement for the kidneys was observed (*r* = 0.96, *P* < 0.0001) with a mean difference of − 6.50 ± 6.76%. This bias between results calculated from SPECT/CT acquisitions inherently aligned has as sole source differences in VOI size and placement between users.

Finally, since in the “single CT” protocol the registration has to be done manually on a SPECT and a CT is not performed simultaneously, we studied the influence of misregistration on a subset of patients. The error in the calculated absorbed dose coming from misregistration depends on the VOI delineation approach used, i.e., manual CT-based delineation or semi-automated SPECT-based segmentation. We chose five SPECT/CTs of different patients for whom the delineation of the kidneys was easily done using the semi-automated SPECT-based segmentation. Dosimetry results obtained with SPECT/CT “inherently aligned” were used as a reference. We repeated the dosimetry calculations while introducing manually a misregistration of 2, 6, 10, and 20 mm in the axial direction between the SPECT and the CT. The relative errors to the kidney dose were 1.8 ± 3.6% (mean ± SD), 5.9 ± 3.4%, 13.0 ± 4.0%, and 27.8 ± 5.3%, respectively, using manual delineation. Using semi-automated SPECT-based segmentation, relative errors were 0.9 ± 1.3%, 0.4 ± 4.9%, 2.8 ± 6.1%, and 3.6 ± 3.3%, respectively. It can be concluded that for small (< 5 mm) misregistration values, its influence on dosimetry values is not significant. Larger values of misregistration are easily detectable by the observer and would prompt manual correction of the registration. In addition, whenever possible, the semi-automated SPECT-based segmentation should be preferred.

## Discussion

The results presented above show that the kidney and bone marrow dosimetry can be reliably done by registration of all but the first post-treatment SPECTs with the CT of the first post-treatment scan. From an operative standpoint, it means that the vast majority of the post-treatment scans can be done on a non-hybrid γ-camera. This result allows for more flexibility in the management of γ-camera time, appreciable in a busy NM center with several cameras, some of which may not be hybrid. Moreover, it opens the possibility of doing these scans in NM centers devoid of hybrid cameras. Although by now most centers performing PRRT are large regional centers equipped with SPECT/CT device, there is no good reason for it since it does not require any specialized equipment. It would be much more desirable and easier to ship the vial of PRRT treatment to small rural centers, similarly to what is done for conventional chemotherapy, than to put the burden of the commutations on the patient and its accompanying person. In this perspective, our work demonstrates that patient’ access to a hybrid SPECT/CT machine should not be the limiting factor to propose him PRRT treatment. An interesting further study would be to assess the reliability of the dosimetry calculations using a single external CT done several months beforehand the onset of treatment that would alleviate completely the need for a hybrid camera in this clinical setup.

From a radiation exposure perspective, this work allows the reduction up to 5/6 = 83% in the non-therapeutic patient exposure. However, the CT radiation is negligible compared to radiation of the treatment. Typically, the radiation dose absorbed by the kidneys following a [^177^Lu]-DOTA-TATE treatment is around 5 Sv [9, 13] versus few mSv [27] for a CT over the abdomen.

**Fig. 7:**
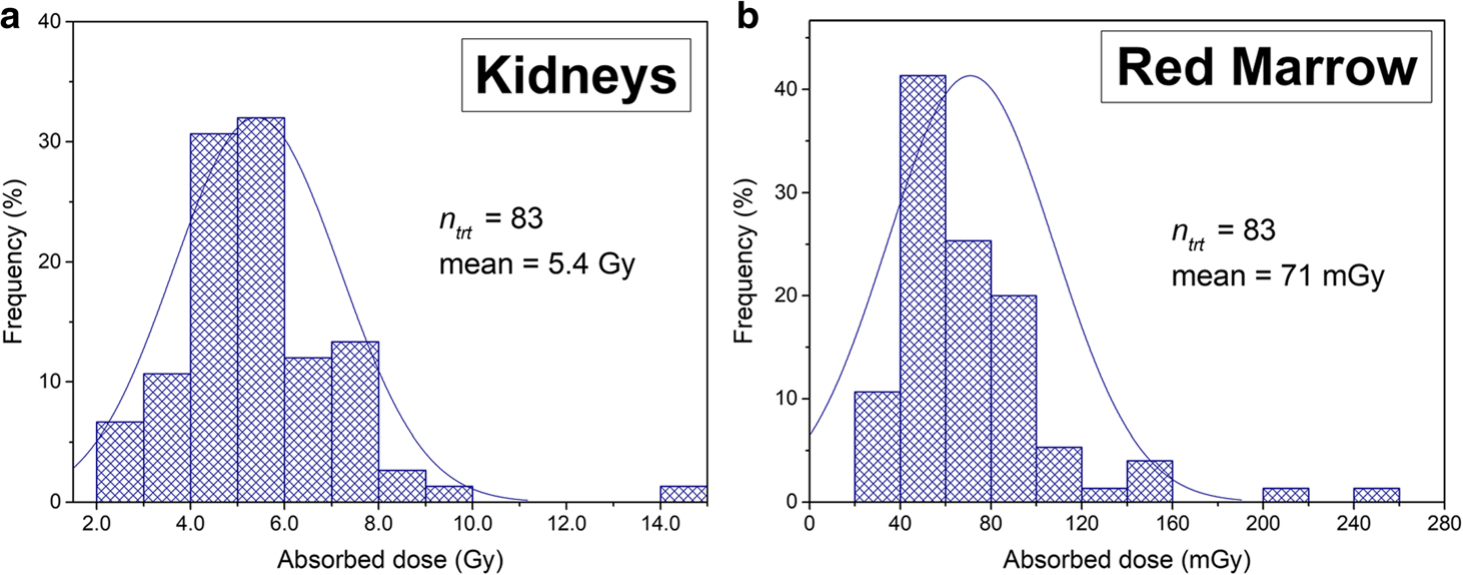
Histogram of the absorbed dose to the (**a**) kidneys and (**b**) bone marrow for 83 therapy cycles given to 24 patients

Our dosimetry data are consistent with previously published works. Figure 7a, b respectively shows the distribution of the dose to the kidneys and bone marrow for the 83 treatments given to the 24 patients included in this work. The mean kidney absorbed dose after a single PRRT treatment of 7.4 GBq was about 5.4 Gy with an absorbed dose ranging from 2 to 14 Gy. This is in agreement with the mean calculated radiation doses in References [7, 13, 28] and with the kidney doses range observed by Sandström et al. [9]. For the bone marrow, the mean kidney absorbed dose per therapy cycle was around 71 mGy which is well below the 100 mGy value found by Sandström et al. [9]. However, the histogram presented in the latter reference presents the absorbed doses for the first therapy cycle only and not for subsequent cycle showing a decrease in the absorbed dose sometimes by more than 50% [9]. Moreover, as said before, here, the mono-exponential fit for the bone marrow residence time calculation was based on radioactivity in the blood at two close time points only, 18 and 25 h post-injection. This certainly led to an underestimation of cumulated activity and therefore of the absorbed dose to this organ.

Finally, it is noteworthy that the influence of the “single CT” protocol on the tumor dosimetry is higher than on the kidney doses. Indeed, a mean relative error of 7.1% between doses calculated with the two protocols with a 95% confidence interval (CI) of − 48.6 and 62.8% has been obtained for tumors over the 83 cycles of treatment. This indicates that differences in tumor dose calculations are spread out over a wider range of values than the kidneys (CI [− 6.9%, 17.5%]). Such deviations are certainly due to lesions misregistration when aligning the first CT with a subsequent SPECT using the kidneys as a reference. However, the clinical relevance of the tumor dosimetry is limited, since it is not part of the clinical management of the patient.

## Conclusions

Absorbed doses to the kidneys and bone marrow were calculated using the “classical” protocol and the new “single CT” protocol over 83 treatments for 24 patients. An excellent agreement between the results calculated from the two protocols was obtained with Pearson’s correlation coefficients *r* = 0.95 and *r* = 0.99 (all *P* < 0.0001) and mean relative differences of 5.30 ± 6.20% and 0.48 ± 4.88% on the cumulative dose for the kidneys and bone marrow, respectively. The management of the patients, i.e., whether or not to stop the treatment because of unsafe absorbed dose to the risk organs using the new protocol, is the same with the two protocols. These results show that dosimetry calculations to critical organs can be done using a single CT registered to serial SPECTs, reducing the need for a hybrid camera.

## Abbreviations

[^177^Lu]-DOTA-TATE: Lutetium-177-DOTA-(Tyr^3^)-octreotate
ALARA: As low as reasonably achievable
CI: Confidence interval
CT: Computed tomography
DTK: Dosimetry toolkit
FOV: Field of view
GE: General Electric
HPLC: High-performance liquid chromatography
IDL: Interactive Data Language
ITLC: Instant thin-layer chromatography
MEGP: Medium-energy general purpose
NETs: Neuroendocrine tumors
OSEM: Ordered subsets expectation maximization
PMT: Photomultiplier tube
PRRT: Peptide receptor radionuclide therapy
PSMA: Prostate-specific membrane antigen
SPECT: Single photon emission computed tomography
VOIs: Volumes of interests
